# Establishment and verification of an osteoporosis risk model in patients with rheumatoid arthritis: a valuable new model

**DOI:** 10.1007/s11657-020-00867-5

**Published:** 2021-01-04

**Authors:** Xiaobin Yan, Zhenhong Xu, Shilin Li, Lisheng Yan, Guorong Lyu, Zecheng Wang

**Affiliations:** 1 Department of Ultrasound, The Second Affiliated Hospital of Fujian Medical University, Quanzhou, 362000 China; 2 Department of Clinical Medicine, Quanzhou Medical College, Quanzhou, 362000 China; 3 Department of Radiology, The Second Affiliated Hospital of Fujian Medical University, Quanzhou, 362000 China

**Keywords:** Rheumatoid arthritis, Osteoporosis, Prediction model, Model verification, 7-joint ultrasound score

## Abstract

**Summary:**

To establish a model for osteoporosis risk in patients with rheumatoid arthritis and validate the model. A newly generated predictive model has been suggested to have good differentiation, calibration, and clinical validity and may be a useful clinical model for predicting osteoporosis in patients with rheumatoid arthritis.

**Purpose:**

To establish a prediction model for osteoporosis risk in patients with rheumatoid arthritis and validate the model internally and externally.

**Methods:**

A total of 270 patients with rheumatoid arthritis who underwent bone mineral density measurement at our hospital from June 2019 to June 2020 were enrolled in the study. The patients were divided into two groups according to their entry time: a training set containing the first 2/3 of the patients (*n* = 180) and a validation set containing the remaining 1/3 of the patients (*n* = 90). Binary logistic regression analysis was used to establish the regression models, and the concordance index (C-index), calibration plot, and decision curve analysis were used to evaluate the prediction model.

**Results:**

Five variables, including age (X1), course of disease (X2), the disease activity score using 28 joint counts (DAS28) (X4), anti-cyclic citrullinated peptide antibody (CCP) (X7), and 7-joint ultrasonic bone erosion (X14), were selected to enter the model. The prediction model is Logit Y = − 12.647 + 0.133X1 + 0.011X2 + 0.754X4 + 0.001X7 + 0.605X14. The model had good differentiation; the C-index in the internal verification was 0.947 (95% CI is 0.932–0.977) and the C-index in the external verification was 0.946 (95% CI is 0.940–0.994). The calibration plot of the model showed excellent consistency between the prediction probability and actual probability. When > 0.483 was taken as the cutoff value for the diagnosis of osteoporosis, the sensitivity, specificity, positive likelihood ratio, negative likelihood ratio, and Jordan index of the model were 90.24%, 87.76%, 7.37, 0.11, and 78.00%, respectively.

**Conclusion:**

A newly generated predictive model has been suggested to have good differentiation, calibration, and clinical validity and may be a useful clinical model for predicting osteoporosis in patients with rheumatoid arthritis.

## Introduction

Rheumatoid arthritis (RA) is a systemic disease with chronic inflammatory joint disease as the main manifestation and can lead to the destruction of cartilage and bone. It is characterized by synovitis, joint destruction, bone loss, and systemic complications [1]. RA is associated with local and systemic inflammation, which can cause bone loss around the joint, bone erosion, osteoporosis, and fractures. Compared with primary osteoporosis, osteoporosis secondary to RA is more likely to lead to fracture [2].

At present, the gold standard for the diagnosis of osteoporosis is bone densitometry. Osteoporosis is a systemic bone disease, and bone loss and deterioration of bone tissue structure lead to bone fragility and increase in fracture susceptibility, especially at the hip, spine, and wrist. Therefore, determination of how to screen groups at high risk for osteoporosis early, comprehensively, and accurately is particularly important. Possible risk factors for osteoporosis in RA include age, sex, low body mass index, disease course, disease activity, CCP, rheumatoid factor (RF), and glucocorticoid use. Musculoskeletal ultrasound is a multiplanar, dynamic, and noninvasive examination method that can be used for dynamic evaluation of disease activities. Research shows that ultrasound and MRI show good correlations in the identification of inflammatory soft tissue and bone erosive bone lesions [3]. Various combinations of joint ultrasound scores can be used to evaluate RA disease activity, but the 7-joint ultrasound score (US7) is the first scoring system combining assessment of synovitis, tenosynovitis, and bone destruction in a comprehensive scoring system [4]. Moreover, US7 is as sensitive to disease changes as the 78-joint score. The US7 includes 7 joints that are most commonly affected in rheumatoid arthritis. Research shows that the joints that are most affected by local erosive bone changes in RA patients are usually the small joints of the hands and feet [5]. Local bone erosion and systemic osteoporosis have a common pathological basis; bone loss is mainly related to inflammation and disease activity, which can aggravate systemic bone loss. With the increase in local bone erosion, the bone density of RA patients decreases, and the osteoporosis incidence increases [6].

To improve the quality of life of patients with rheumatoid arthritis and detect osteoporosis early, it is necessary to develop a valid and reliable model to predict osteoporosis in patients with rheumatoid arthritis. Therefore, this study aimed to establish and verify a reliable prediction model for osteoporosis that could play an important role in the early clinical detection of osteoporosis in patients with rheumatoid arthritis.

## Materials and methods

### General information

A total of 270 patients with rheumatoid arthritis were recruited from the Department of Rheumatology and Immunology at our hospital from June 2019 to June 2020. According to the gold standard bone mineral density measurement, the subjects were divided into an osteoporosis group and a group without osteoporosis. There were 84 patients in the osteoporosis group, including 63 females and 21 males; the average age was 60.51 ± 9.95 years old, the course of disease was 36–120 months, and the duration of medication was 15–78.5 months. In the group without osteoporosis, there were 96 patients, including 76 females and 20 males; the mean age was 50.21 ± 9.40 years, the course of disease was 7.25–78.75 months, and the duration of medication was 3.25–40 months. There were 180 patients in the training set, including 139 females and 41 males; the average age was 55.02 ± 10.93 years old, the course of disease was 13.75–120 months, and the duration of medication was 6.25–60 months. There were 90 patients in the validation set, including 72 females and 18 males; the average age was 53.87 ± 10.72 years old, the course of disease was 25–120 months, and the duration of medication was 9.50–60.50 months. The research protocol was examined and approved by the Ethics Committee of the Second Affiliated Hospital of Fujian Medical University. All participants provided informed consent.

### Diagnostic criteria

All the research subjects were classified in accordance with the classification criteria for rheumatoid arthritis established by the American College of Rheumatology (ACR)/European Union Against Rheumatism (EULAR) in 2010[7]. The diagnostic criteria for osteoporosis used in this study are in line with the diagnostic criteria for osteoporosis in the China Guidelines for the Diagnosis and Treatment of Senile Osteoporosis (2018) [8].

### Exclusion criteria

Patients < 18 years old with a BMI < 18.5; patients with other immune and endocrine diseases, kidney diseases, severe liver and kidney dysfunction, blood system diseases, hysterectomy, premature menopause history (< 45 years old) and other diseases that affect changes in bone density; congenital joint dysplasia, joint trauma, and joint replacement surgery history; pregnancy or lactation; heavy smokers and drinkers (more than 3 units/day); and patients with long-term use of drugs that cause osteoporosis (including long-term (cumulative dose > 2 years) or high-dose (> 7.5 mg/day) glucocorticoid use).

### Instruments and methods

#### Instruments

The ultrasonic instrument used was China Mindray 7 ultrasonic diagnostic instrument with a high-frequency linear array probe (frequency 4–15 MHz); the skeletal muscle system imaging mode was selected. The bone mineral density was examined using a Hologic Discovery dual-energy X-ray densimeter.

### Methods

#### Laboratory examination

The results of the detection of C-reactive protein (C-RP), erythrocyte sedimentation rate (ESR), rheumatoid factor (RF), and anti-cyclic citrullinated peptide antibody (CCP) were recorded for all patients (normal values C-RP, 0–8 mg/L; ESR, ≦ 20 mm/h; RF, 0–30 IU/ml; CCP, 0–25 RU/ml).

#### RA activity scoring method

All subjects were scored according to the DAS28 scoring method. Calculation formula: DAS28 = [0.56 × sqrt(t28) + 0.28 × sqrt(sw28)+

### 0.70 × Ln (ESR)] × 1.08 + 0.16.

#### Ultrasonic examination

The ultrasonic operation steps were carried out according to the guidelines for the high-frequency ultrasonic examination of muscle joints formulated by the European Rheumatism Union. The wrist joint (Wri), the second and third metacarpophalangeal joints (MCP2, MCP3), the second and third proximal interphalangeal joints (PIP2, PIP3), and the second and fifth metatarsophalangeal joints (MTP2, MTP5) of the affected dominant limb (on the side of the body with more severe symptoms and signs) were detected with a 7-joint ultrasound scoring system. When examining the Wri, MCP2, MCP3, PIP2, and PIP3 joints, the patient sits directly opposite the examining physician and keeps their wrist and elbow relaxed and lying flat on the examination table (if elderly or injured patients cannot maintain the above posture, they can lie flat on the examination bed with their upper arms on both sides of the body).When examining the MTP2 and MTP5 joints, the patient takes a supine or sitting position to extend or bend the knee joint by 45° to keep the ankle joint in a natural or resting position. Grayscale ultrasound (GSUS) tendon tenosynovitis and bone erosion were evaluated according to a binary classification score, with “none” scored as 0 and “yes” scored as 1. GSUS was evaluated according to a semiquantitative score to evaluate synovitis: no synovial hyperplasia: 0 points; the synovial membrane was slightly thickened, and the highest point was lower than the level of the joint cavity connection: 1 point; the synovial membrane was moderately thickened, and the highest point was close to the level of the joint cavity connection: 2 points; the synovial membrane was severely thickened, and the highest point exceeded the level of the joint cavity connection: 3 points. Power doppler ultrasound (PDUS) was used with a semiquantitative method to evaluate the blood flow signals in the hyperplastic synovium and tendon sheath: no obvious blood flow signal: 0 points; ≦ 3 dots or 2 dots and 1 linear blood flow signal: 1 point; > 3 punctate blood flows or blood flows fused into slices but with ≦ 50% lesion area: 2 points; dendritic and reticular blood flow signals can be found in the lesion, with the range exceeding 50% of the lesion: 3 points. According to the scores of GSUS synovitis, PDUS synovitis, GSUS tenosynovitis, PDUS tenosynovitis, and ultrasonic bone erosion in each joint, the individual scores of the 7 joints were calculated, and the individual scores were summed to determine the total US7 score.

##### Bone mineral density examination

All patients were measured by Hologic Discovery A dual-energy X-ray absorptiometry. The bone mineral densities of lumbar spine 1-4, femoral neck, total hip, and distal radius 1/3 were measured by a conventional DXA measurement method. The definition of osteoporosis was defined as the BMD value < 2.5 T-score at any of the 3 measured locations (i.e., lumbar spine 1–4, femoral neck, total hip).

### Statistical analysis

SPSS statistics v20 (IBM Corp, Los Angeles, CA, USA) and R statistical software (version 4.0.2; http://www.R-project.org/) were used to analyze the data. When the measurement data conformed to the normal distribution, they were summarized as the mean ± standard deviation; the nonnormally distributed data were summarized using the median (M, P_25_-P_75_). The independent sample *t* test was used for comparison of the means between two groups conforming to a normal distribution, and the Mann-Whitney *U* test was used for comparison of nonnormally distributed data. Count data are expressed as percentages and ratios, and the chi-square test was used for comparison. Spearman correlation analysis was performed to determine the correlations between the medication time, age, disease course, US7 system scores, CRP, ESR, RF, CCP, DAS28, and the different bone density groups. A prediction model was established in the training set. In the univariate analysis, the variables (*p < 0.1*) that may be related to rheumatoid arthritis osteoporosis were included in the binary logistic regression analysis. The final prediction model was constructed with variables significant in the univariate analysis (*p* < 0.05) and variables considered to be clinically relevant. Using the nomogram function in the rms package in R statistical software, a nomogram for predicting the possibility of osteoporosis in patients with rheumatoid arthritis was established. To reflect the predictive model’s ability to accurately distinguish patients with osteoporosis from patients without osteoporosis, an ROC curve was drawn with the help of the pROC package in R statistical software, and the optimal cutoff value was calculated. The AUC value represents the discrimination capacity of the model. The greater the AUC value, the better the discrimination of the model. To evaluate the consistency between the predicted risk and the actual risk, a calibration plot was drawn using the val.prob function in the rms package in R statistical software; the closer the calibration line of the model is to the standard line, the better the calibration degree of the model is. The dca package in R statistical software was used to draw the clinical decision curve to reflect the clinical effectiveness of the model. *p* < 0.05 indicates that the difference is statistically significant.

## Results

### Comparison of demographic characteristics, laboratory values and ultrasonography in patients with rheumatoid arthritis

There was no significant difference between the training set and the verification set in terms of the demographic characteristics, laboratory values, or ultrasonography (Table 1).

**Table 1:** The difference in training set and the verification set in terms of the demographic characteristics, laboratory values, or ultrasonography

Variables	The training set (*n* = 180)	The validation set (*n* = 90)	*p*
Age(years)	55.02 ± 10.93	53.87 ± 0.72	0.413
Gender(female/male)	139/41	72/18	0.603
Disease duration(months)	(13.75–120.00	(25.00–120.00	0.092
Treatment duration(months)	(6.25–60.00)	( (9.50–60.50)	0.650
DAS-28	(2.77–5.18 )	(2.89–5.42)	0.472
CRP(mg/L)	( (2.88–25.28)	(2.79–23.20)	0.635
ESR (mm/H)	(20.25–67.25)	( (18.50–65.75)	0.732
RF (IU/mL)	(20.00–226.75)	(22.63–19.25)	0.067
CCP (RU/mL)	(1 (12.01–644.08)	( (19.99–59.76)	0.184
Synovitis score in GSUS	(11.00–19.00)	(13.00–20.00)	0.066
Synovitis score in PDUS	(1.25–8.00)	( (2.00–8.00)	0.723
Tenosynovitis score in GSUS	(0–2)	(0–2)	0.629
Tenosynovitis score in PDUS	(0–1)	(0–1)	0.929
7-joint ultrasonic erosions score	(0–3)	(0–3)	0.832
Total US7 score	( (13.00–32.00)	(14.00–34.25)	0.340

### Correlation analysis of osteoporosis-related factors in the training set

In 180 patients with RA, DAS28 (*r* = 0.629, *p* < 0.001), 7-joint ultrasonic bone erosion (*r* = 0.634, *p* < 0.001), and the total US7 score (*r* = 0.624, *p* < 0.001) were positively correlated with osteoporosis in RA patients. Age (*r* = 0.454, *p* < 0.001), CRP (*r* = 0.481, *p* < 0.001), ESR (*r* = 0.479, *p* < 0.001), CCP (*r* = 0.409, *p* < 0.001), synovitis score on GSUS (*r* = 0.514, *p* < 0.001), synovitis score on PDUS (*r* = 0.574, *p* < 0.001), tenosynovitis score on GSUS (*r* = 0.597, *p* < 0.001), and tenosynovitis score on PDUS (*r* = 0.503, *p* < 0.001) were moderately correlated with osteoporosis in RA patients. Disease duration (*r* = 0.346, *p* < 0.001), RF (*r* = 0.372, *p* < 0.001*)*, and treatment duration (*r* = 0.326, *p* < 0.001) were weakly positively correlated with the severity of osteoporosis in RA patients.

### Univariate analysis of osteoporosis-related factors in the training set

The variables suspected to predict osteoporosis were analyzed between patients with osteoporosis and patients without osteoporosis in the training set, and the results are shown in Table 2. Except for sex, there were differences between the osteoporosis and nonosteoporosis patients, and the differences were statistically significant.

**Table 2:** Results of the variables suspected to predict osteoporosis between patients with osteoporosis and patients without osteoporosis in the training set

Variables	Osteoporosis (*n* = 84)	Without osteoporosis (*n* = 96)	*p*
Age(years)	(60.51 ± 9.95)	(50.21 ± 9.40)	< 0.001
Gender(female/male)	63/21	76/20	0.506
Disease duration(months)	(36.00–120.00)	(7.25–78.75)	< 0.001
Treatment duration(months)	(15.00–78.50)	(3.25–40.00)	< 0.001
DAS-28	(4.08–6.13)	(2.30–3.53)	< 0.001
CRP(mg/L)	(6.96–36.60)	(1.89–10.55)	< 0.001
ESR (mm/H)	(35.00–89.75)	(14.25–42.00)	< 0.001
RF (IU/mL)	(27.50–523.00)	(20.00–102.00)	< 0.001
CCP (RU/mL)	(154.82–1047.61)	(7.00–383.23)	< 0.001
Synovitis score in GSUS	(15.00–21.00)	(9.00–15.75)	< 0.001
Synovitis score in PDUS	(5.00–10.00)	(1.00–4.00)	< 0.001
Tenosynovitis score in GSUS	(0–3)	(0–0)	< 0.001
Tenosynovitis score in PDUS	(0–2)	(0–0)	< 0.001
7-joint ultrasonic erosions score	(2–4)	(0–0)	< 0.001
Total US7 score	(22.25–38.00)	(10.00–21.00)	< 0.001

### Osteoporosis prediction model developed in the training set

Age, disease duration, treatment duration, DAS28, CRP, ESR, CCP, RF, synovitis score on GSUS, synovitis score on PDUS, tenosynovitis score on GSUS, tenosynovitis score on PDUS, 7-joint ultrasonic bone erosion, and total US7 scores were designated as variables X1-X14, respectively. Variables X1-X14 were all significant factors identified through single-factor analysis and were included in the binary logistic regression analysis. Five variables, namely age (X1), disease course (X2), DAS28 (X4), CCP (X7), and 7-joint ultrasonic bone erosion (X14), were statistically significant in the constructed model (Table 3). The predictive model with five significant independent variables was logit Y = − 12.647 + 0.133X1 + 0.011X2 + 0.754X4 + 0.001X7 + 0.605X14. The overall accuracy rate of the model was 88.3%, and the accuracy rate of predicting osteoporosis with the model was 86.9%.

**Table 3:** Five variables, namely age (X1), disease course (X2), DAS28 (X4), CCP (X7), and 7-joint ultrasonic bone erosion (X14), were statistically significant in the constructed model

Variables	B score	Wald score	*p*	OR score	95%CI
Age	0.133	17.409	< 0.001	1.143	1.107~1.127
Disease duration	0.011	5.382	0.02	1.011	1.002~1.020
DAS28	0.754	11.293	0.001	2.126	1.369~3.300
CCP	0.001	5.412	0.02	1.001	1.000~1.002
7-joint ultrasonic erosions score	0.605	11.508	0.001	1.832	1.291~2.599

### Internal and external validation of the model

**Fig. 1:**
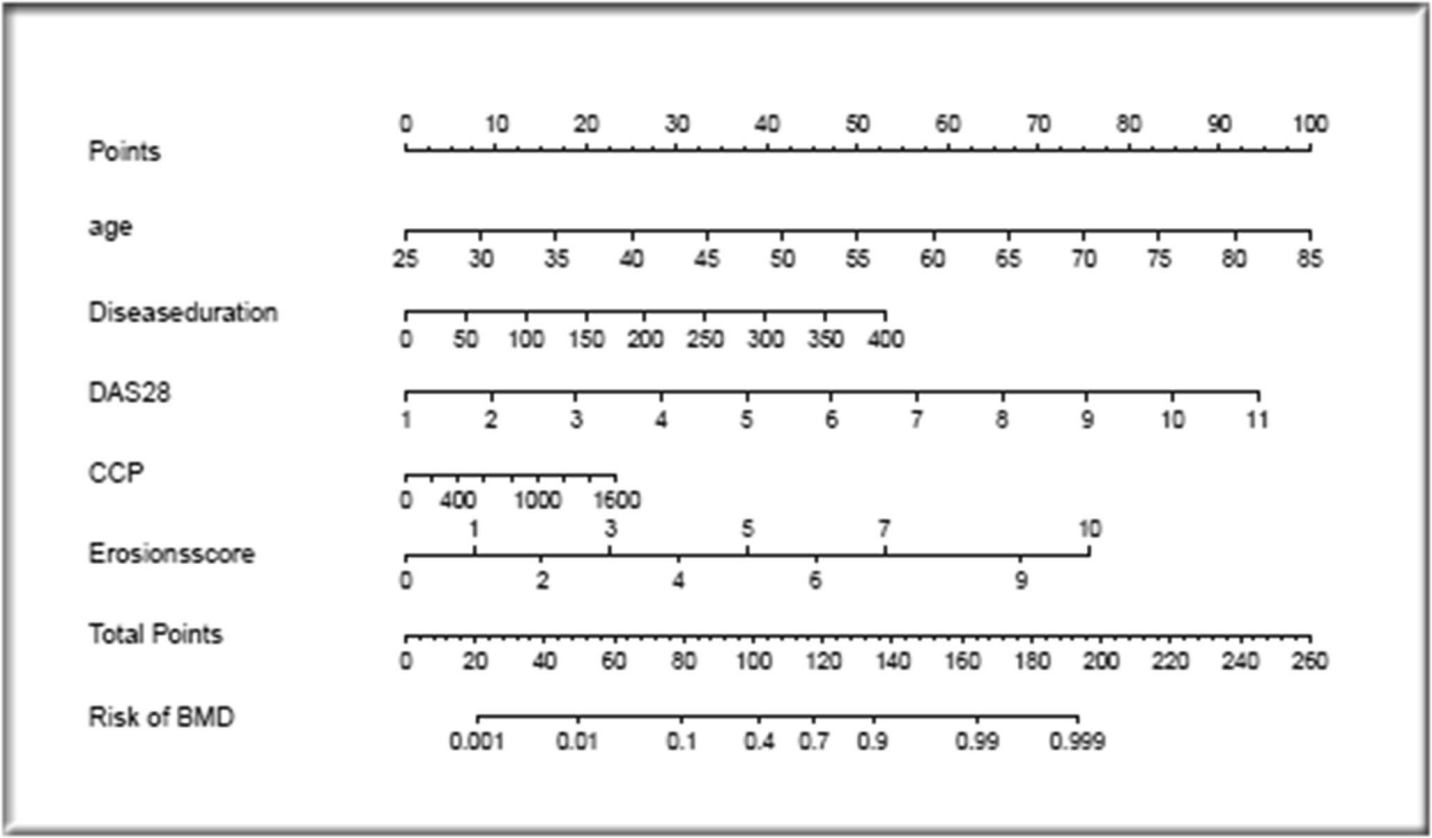
Nomogram for predicting osteoporosis in patients with rheumatoid arthritis

**Fig. 2:**
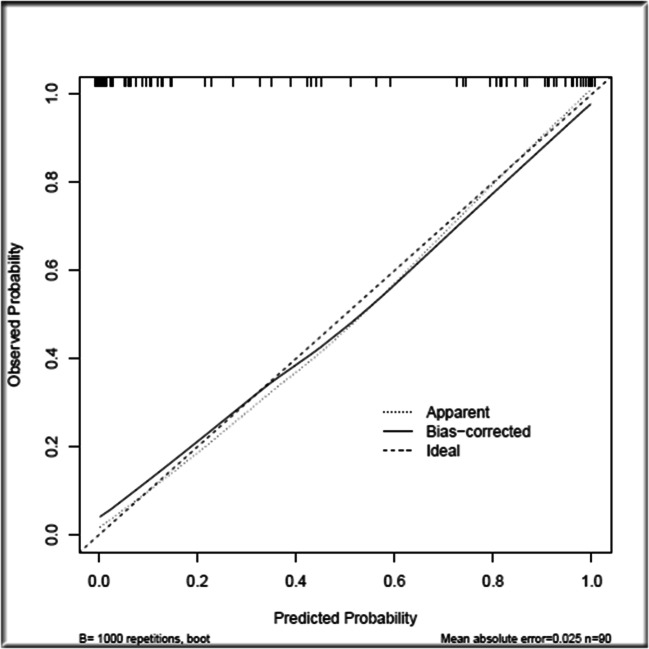
The calibration plot of the novel nomogram in the verification set. The logistic correction curve (solid black line) is close to the ideal reference line (solid gray line), which indicates that the nomogram performed well in the verification set

**Fig. 3:**
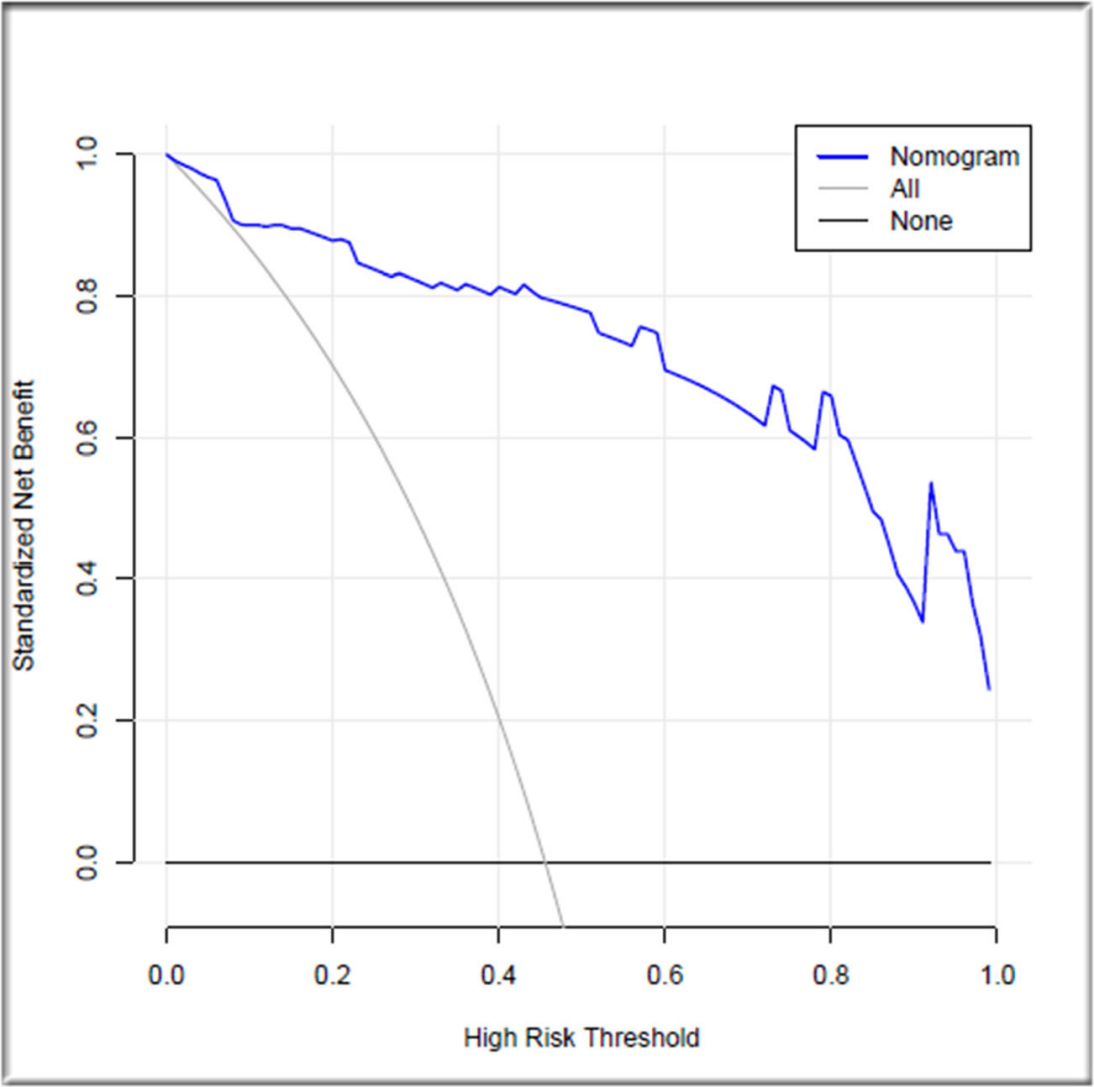
Decision curve for evaluating the clinical effectiveness of the prediction model. In the decision curve, the abscissa is the threshold probability, the ordinate is the net benefit, and there are two extreme lines besides the model curve (gray horizontal line indicates that all samples are negative, that is, the osteoporosis probability is less than the threshold probability, and none of the patients had osteoporosis, so the overall net benefit is 0; the gray curve indicates the opposite situation, that is, all of the patients had osteoporosis, and the net benefit is negative). The thick blue line indicates the prediction model

Based on the binary logistic regression model, a new nomogram was established (Fig. 1). According to the visual graph, each predictor was assigned a score, and then the scores are added to obtain the total score. Finally, the corresponding osteoporosis risk is read according to the total score and used to help formulate the clinical treatment strategy. The age variable has the largest absolute effect (100 points), and the remaining variables are scored according to the weight of each effect. Then, by adding the scores for all of the model variables and comparing the total score to the scale in the nomogram, the risk of osteoporosis can be determined. When the model was verified internally, the C-index value for predicting osteoporosis was 0.947 (95% CI 0.932~0.977). The C-index value for predicting osteoporosis predicted on external verification was 0.946 (95% CI 0.940~0.994). The calibration graph shows good agreement between the deviation-corrected prediction and the ideal reference line in the training set and the verification set (Fig. 2). When > 0.483 was taken as the cutoff value for the diagnosis of osteoporosis, the sensitivity, specificity, positive likelihood ratio, negative likelihood ratio, and Jordan index of the model were 90.24%, 87.76%, 7.37, 0.11, and 78.00%, respectively. According to the Hosmer-Lemeshow test, the *p* values of the training group and verification group were 0.929 and 0.902, respectively, which indicates that the model was in good agreement with the observed data. It is suggested that this model is suitable for use in rheumatoid arthritis patients, and it can be used to judge whether further bone mineral density measurement is needed according to the nomogram scoring system. In the decision curve, the model curve in the training set is notably better than the two extreme lines, suggesting that the overall net benefit in the population is good; the verification set also performed well (Fig. 3).

## Discussion

RA is a chronic inflammatory disease characterized by continuous inflammation of the synovium, joint destruction, bone loss, and systemic complications [9]. Bone changes in rheumatoid arthritis include periarticular bone erosion, periarticular bone loss, and systemic osteoporosis [10].

There are many possible risk factors for osteoporosis in RA patients, such as age, female sex, low body mass index, glucocorticoid use (daily dose ≥ 7.5 mg), long disease course, high activity, and CCP [11].

Our study shows that the osteoporosis group was older and has a longer disease course than the group without osteoporosis; age and disease course were independent risk factors for predicting osteoporosis. Research by Tong shows that age is a risk factor for osteoporosis in RA patients [12]. The risk of vertebral fracture in RA patients increases by 7.2% for every 1 year of increase in age. Research by Gauri also shows that patients with a longer disease course and higher activity are more likely to suffer from osteopenia and osteoporosis [13]. Previous studies showed that the use of glucocorticoids and other drugs was an independent risk factor for osteoporosis [14]. Interestingly, our research results showed that compared with the osteoporosis group and the group without osteoporosis, the osteoporosis group had a longer treatment duration, and there was a weak correlation between the treatment duration and osteoporosis (*r* = 0.346, *p* < 0.001). The reason for this may be that we have excluded long-term (cumulative dose > 2 years) and high-dose (> 7.5 mg/day) glucocorticoid patients, and many studies show that short-term, low-dose glucocorticoid therapy can stabilize the bone mineral density in early RA with high disease activity [15].

DAS28 is an important way to evaluate the disease activity of RA. Our research shows that the disease activity of the two groups of patients was different, with the disease activity of osteoporosis group being the higher. DAS28 is an independent predictor of osteoporosis, and disease activity was strongly correlated with osteoporosis (*r* = 0.629, *p* < 0.001). This indicates that there was a correlation between disease activity and bone loss. Bone mineral density loss occurs in the early stage of RA and increases with the increase of disease activity, which is consistent with the research viewpoints of many scholars [16, 17]. Research shows that the CRP and ESR values of the osteoporosis groups were higher than the non-osteoporosis group, and there was a moderate correlation with osteoporosis, but CRP and ESR were not independent predictors of osteoporosis; the research of Tomizawa and other scholars also confirm this point [18].

There are many kinds of autoantibodies in the sera of RA patients, with the most common being CCP and RF. In our study, the CCP were different between the osteoporosis and the group without osteoporosis. Many studies have shown that CCP is associated with local and systemic bone mineral density reduction and osteoclast-mediated bone resorption in RA patients [19]. For early RA patients, when CCP is positive, high-frequency ultrasound is more likely to find articular cartilage destruction and bone erosion changes [20]. Our research shows that CCP is a predictor of osteoporosis. Research by Tomizawa showed that CCP is a risk factor not only for joint destruction in RA patients but also for bone loss. It is worth mentioning that although RF is not a predictor of osteoporosis, there were differences between the osteoporosis and the group without osteoporosis, and there was correlation with osteoporosis; the reason may be that the relationship between RF and BMD loss is obviously dose-dependent, and a significant difference was only observed in patients with high levels because high levels of RF enhance the relationship between CCP and bone loss [21].

All indexes of the US7 score were significantly different between the osteoporosis group and the group without osteoporosis. 7-joint ultrasonic bone erosion (*r* = 0.634, *p* < 0.001) and US7 total score (*r* = 0.624, *p* < 0.001) had a strong positive correlation with osteoporosis. Gong’s research on the correlation between systemic osteoporosis and local bone erosion in rheumatoid arthritis patients in China shows that osteoporosis is the early manifestation of RA bone erosion; it has been confirmed that RA is related to a high risk of osteoporosis, and whole body bone density is related to local bone erosion in rheumatoid arthritis patients in China. With the increase in local bone erosion, the bone density of RA patients decreases, and the incidence of osteoporosis increases [22]. Bone erosion is generally regarded as irreversible, and it is the key result of inflammatory rheumatism, which is related to the severity of disease and deterioration of function [23]. Elshahaly used X-ray to detect hand and foot bone erosion and study its relationship with hip bone density. The results showed that the hip bone density of rheumatoid arthritis patients with bone erosion was significantly lower than that of patients without bone erosion. The results suggested that focal bone loss in RA was closely related to systemic bone loss [24]. Numerous studies have shown that the joints most frequently invaded by rheumatoid arthritis are the facet joints; the 2nd metacarpophalangeal joint, 3rd metacarpophalangeal joint, 5th metacarpophalangeal joint, 2nd metatarsophalangeal joint, 5th metatarsophalan-geal joint, and the joints detected by the 7-joint ultrasonic score method are the joints most prone to rheumatoid arthritis bone erosion [25, 26]. Compared with X-ray detection of hand and foot bone erosion in patients with rheumatoid arthritis, ultrasound detection has the advantage no radioactivity and real-time results, and ultrasound can also detect bone and joint from palmar, dorsal, medial, and lateral angles on the long-axis and short-axis tangent plane. Roux’s research also proved that ultrasound is a reliable way to evaluate bone erosion. The number of RA patients with bone erosion detected by ultrasound is approximately twice that of X-ray examination, especially in early RA [27].

Many studies have used multiple logistic regression analysis to analyze the risk factors for osteoporosis in RA patients. The results showed that age, course of disease, CCP, and DAS28 were risk factors for osteoporosis in RA patients, which was similar to the results of our study [28, 29]. With regard to the prediction model of rheumatoid arthritis osteoporosis, Meng’s research used the Asian osteoporosis self-assessment tool (OSTA) to explore the value of the OSTA index in predicting osteoporosis in elderly patients with rheumatoid arthritis in China [30]. The results of binary logistic regression analysis showed that the Sharp score was an independent risk factor for RA-induced osteoporosis, while the OSTA index was the only protective factor. Aizer’s research used stepwise logistic regression analysis to determine the independent predictors of osteoporosis [31]. The results showed that the independent predictors were age, female sex, fracture history, steroid use, and the doctor’s assessment of RA activity. Although two scholars have performed regression analysis on rheumatoid arthritis osteoporosis and identified the risk factors for osteoporosis, they have not verified the prediction model. Therefore, our study not only established a prediction model of rheumatoid arthritis osteoporosis but also verified the model. The C-index value for predicting osteoporosis was 0.947 (95% CI = 0.932~0.977) in the internal validation and 0.946 (95% CI = 0.940~0.994) in the external validation. The calibration plot showed good consistency between the deviation corrected prediction and the ideal reference line in both the training set and the verification set. In the decision curve, the model curve in the training set was notably better than the two extreme lines, suggesting that the overall net benefit of the population was good; the verification set also performed well. The verification results show that the model has not only good discrimination and calibration ability but also good clinical applicability.

This study has several limitations. First, this was a single-center study. Although we have verified the model internally and externally in independent cohorts in the same center, the conclusion should be cautious; we will continue to collect data from other centers for further verification. Second, the sample size of this study was small, so we will continue to collect cases and conduct multicenter research and verification in the future. Third, excluding patients with long-term use of drugs that cause osteoporosis (including long-term (cumulative dose > 2 years) or high-dose (> 7.5 mg/day) glucocorticoid use) will influence our results and limit their applicability to other populations. Fourth, the prevalence of rheumatoid arthritis was different from those in other ethnic groups. Therefore, this nomogram requires validation in other ethnic groups’ cohort and may require modification prior to its general use.

In summary, a prediction model based on age, course of disease, DAS28, CCP, and 7-joint ultrasonic bone erosion has good discrimination, calibration, and clinical effectiveness and may become a useful clinical model for predicting osteoporosis risk in patients with rheumatoid arthritis.

## References

[1] Sparks JA (2019). Rheumatoid arthritis [J]. Ann Intern Med.

[2] Heinlen L, Humphrey MB (2017). Skeletal complications of rheumatoid arthritis [J]. Osteoporos Int.

[3] Fukae J, Kon Y, Henmi M, et al. Change of synovial vascularity in a single finger joint assessed by power doppler sonography correlated with radiographic change in rheumatoid arthritis: comparative study of a novel quantitative score with a semiquantitative score [J]. Arthritis Care Res (Hoboken),2010,62(5):657-63. 10.1002/acr.20110 20191472

[4] Ohrndorf S, Fischer IU, Kellner H, et al. Reliability of the novel 7-jointultrasound score: results from an inter- and intraobserver study performed byrheumatologists [J]. Arthritis Care Res (Hoboken),2012,64(8):1238-43. 10.1002/acr.21679 22438306

[5] El-Gohary RM, Ahmed Mahmoud AA, Khalil A (2019). Validity of 7-joint versus simplified 12-joint ultrasonography scoring systems in assessment of rheumatoid arthritis activity [J]. J Clin Rheumatol.

[6] Yamamoto Y, Turkiewicz A, Wingstrand H (2015). Fragility fractures in patients with rheumatoid arthritis and osteoarthritis compared with the general population [J]. J Rheumatol.

[7] Aletaha D, Neogi T, Silman AJ (2010). 2010 Rheumatoid arthritis classification criteria: an American college of rheumatology/European league against rheumatism collaborative initiative [J]. Arthritis & Rheumatism.

[8] Ma YZ, Wang YP, Liu Q, et al.2018 China guideline for diagnosis and treatment of senile osteoporosis [J].Chin J Osteoporos,2018,24(12):1541-1567.

[9] Llorente I, Merino L, Ortiz AM (2017). Anti-citrullinated protein antibodies are associated with decreased bone mineral density: baseline data from a register of early arthritis patients [J]. Rheumatol Int.

[10] Heinlen L, Humphrey MB (2017). Skeletal complications of rheumatoid arthritis [J]. Osteoporos Int.

[11] Sargın G, Köse R, Şentürk T (2019). Relationship between bone mineral density and anti-citrullinated protein antibody and rheumatoid factor in patients with rheumatoid arthritis [J]. Eur J Rheumatol.

[12] Tong JJ, Xu SQ, Zong HX (2020). Prevalence and risk factors associated with vertebral osteoporotic fractures in patients with rheumatoid arthritis [J]. Clin Rheumatol.

[13] Gauri LA, Fatima Q, Diggi S (2017). Study of bone mineral density (BMD) in patients with rheumatoid arthritis and its corelation with severity of the disease [J]. J Assoc Physicians India.

[14] Blavnsfeldt AG, de Thurah A, Thomsen MD (2018). The effect of glucocorticoids on bone mineral density in patients with rheumatoid arthritis: a systematic review and meta-analysis of randomized, controlled trials [J]. Bone.

[15] Adami G, Saag KG (2019). Osteoporosis pathophysiology, epidemiology, a screening in rheumatoid arthritis [J]. Curr Rheumatol Rep.

[16] Zeng TT, Tian YJ, Tan LM, et al. Risk factors analysis of osteoporosis in rheumatoid arthritis [J].Chin J Osteoporos,2019,25(01):74-78 + 84.

[17] Ahmad HA, Alemao E, Guo Z (2018). Association of low bone mineral density with anti-citrullinated protein antibody positivity and disease activity in established rheumatoid arthritis: findings from a US observational cohort [J]. Adv Ther.

[18] Tomizawa T, Ito H, Murata K (2019). Distinct biomarkers for different bones in osteoporosis with rheumatoid arthritis [J]. Arthritis Res Ther.

[19] Orsolini G, Caimmi C, Viapiana O (2017). Titer-dependent effect ofanti-citrullinated protein antibodies on systemic bone mass in rheumatoid arthritis patients [J]. Calcif Tissue Int.

[20] Yang J, Shao Q, Wu J. Correlation between high-frequency ultrasonography of patients with early rheumatoid arthritis and anti-CCP antibody [J]. Medicine (Baltimore),2019,98(6):e14083. 10.1097/MD.0000000000014083 PMC6380833 30732128

[21] Bugatti S, Bogliolo L, Vitolo B (2016). Anti-citrullinated protein antibodies and high levels of rheumatoid factor are associated with systemic bone loss in patients with early untreated rheumatoid arthritis [J]. Arthritis Res Ther.

[22] Gong X, Xu SQ, Tong H, et al. Correlation between systemic osteoporosis and local bone erosion with rheumatoid arthritis patients in Chinese population [J]. Rheumatology (Oxford),2019,kez042. 10.1093/rheumatology/kez042 30847477

[23] Coury F, Peyruchaud O, Machuca-Gayet I (2019). Osteoimmunology of bone loss in inflammatory rheumatic diseases [J]. Front Immunol.

[24] Elshahaly MH, Gad KA (2020). The utility of radiographic focal erosions of hands or feet in predicting DXA-defined osteoporosis of the hip in patients with rheumatoid arthritis [J]. Curr Rheumatol Rev.

[25] Zayat AS, Ellegaard K, Conaghan PG (2015). The specificity of ultrasound detected bone erosions for rheumatoid arthritis [J]. Ann Rheum Dis.

[26] Tan YK, Li H, Allen JC (2020). Extended 36-joint sonographic examination: what it reveals about bone erosions in patients with rheumatoid Arthritis [J]. J Clin Ultrasound.

[27] Roux C, Gandjbakhch F, Pierreisnard A (2019). Ultrasonographic criteria for the diagnosis of erosive rheumatoid arthritis using osteoarthritic patients as controls compared to validated radiographic criteria [J]. Joint Bone Spine.

[28] Wysham KD, Shoback DM, Imboden JB (2018). Association of high anticyclic citrullinated peptide seropositivity and lean mass index with low bone mineral density in rheumatoid arthritis [J]. Arthritis Care Res (Hoboken).

[29] Wang Y, Geng Y, Deng XR, et al. Relationship between wrist bone mineral density and synovitis, erosions by ultrasonography in female rheumatoid arthritis patients [J]. Jounal of Peking University (Health Sciences),2015,47(05):774-780. 26474614

[30] Tong H, Zong HX, Xu SQ (2019). Osteoporosis self-assessment tool as a screening tool for predicting osteoporosis in elderly Chinese patients with established rheumatoid arthritis [J]. J Clin Densitom.

[31] Aizer J, Reed G, Onofrei A (2009). Predictors of bone density testing in patients with rheumatoid arthritis [J]. Rheumatol Int.

